# Editorial for Recent Advances in Autoimmune Rheumatic Diseases: 2nd Edition

**DOI:** 10.3390/medicina61111889

**Published:** 2025-10-22

**Authors:** Diana Mieliauskaitė

**Affiliations:** Department of Personalised Medicine, State Research Institute Centre for Innovative Medicine, Santariškių St. 5, LT-08406 Vilnius, Lithuania; diana.mieliauskaite@imcentras.lt

This Special Issue of *Medicina*, entitled “Recent Advances in Autoimmune Rheumatic Diseases”, is dedicated to the state of the art in autoimmune rheumatic diseases, with the expectation that it will be highly beneficial in clinical practice and enhance public health, and provide the latest information on innovations in the field of autoimmune rheumatic diseases.

Autoimmune rheumatic diseases (ARD) are a major global health problem with increasing prevalence. In addition, these diseases are linked to high morbidity, mortality, and disability rates. Autoimmune rheumatic diseases do not have diagnostic criteria and are guided by classification criteria, so the confirmation of the diagnosis is often delayed. These diseases as highly heterogeneous diseases involve complex pathophysiological mechanisms, resulting in variations in treatment response among individuals [1,2]. This heterogeneity underscores the need for individualized and precise treatment strategies and encourages patient-centered collaboration between clinicians and researchers.

We present the latest research advances in rheumatoid arthritis, vasculitis, SLE, and other autoimmune rheumatic diseases in this Special Issue. In parallel, the latest research advances in the field of autoimmune rheumatic diseases are summarized in reviews. Therefore, I would like to briefly review the contents of this Issue.

The introduction of Janus kinase inhibitors (JAKIs) for the treatment of rheumatoid arthritis (RA) has significantly changed treatment options. When using Janus kinase inhibitors (JAKIs), it is important to consider the safety of the drug, especially the risk of cardiovascular disease and cancer, and so additional studies are conducted in real clinical conditions. Priora M et al. evaluated the long-term efficacy of Janus kinase inhibitor Tofacitinib (TOFA) and predictors of responses to treatment in two RA patient subpopulations stratified according to different cardiovascular risk profiles. Summarizing the results, the authors conclude that tofacitinib showed similar efficacy in RA patients with both high- and low-risk cardiovascular subgroups, and disease activity decreased significantly both after six months and after one year. TOFA remained an effective treatment for all patients, and remission or LDA rates were reasonable despite safety considerations regarding the risk of cardiovascular events. Age had a negative impact on treatment response. This highlights the role of immunosenescence in the treatment of RA. The study results confirm the use of TOFA as an individualized approach to RA treatment, emphasizing the importance of carefully assessing cardiovascular and age-related risk factors when making clinical decisions [3].

The relationship between the use of rehabilitation services (RS) and changes in inflammatory responses in patients with RA has not yet been sufficiently investigated, although the effectiveness of rehabilitation services (RS) is supported by evidence. Huang HJ et al. sought to evaluate changes in inflammatory markers before and after long-term rehabilitation. The authors conclude that integrating RS into routine care may be beneficial for controlling inflammation in RA patients. Further studies should be conducted using randomized controlled trials to confirm the effectiveness of RS [4].

In chronic autoimmune rheumatic diseases such as rheumatoid arthritis, self-efficacy is an important element of successful disease management. However, there is little information available on self-efficacy and its influencing factors among RA patients. Hsiao I-Y et al. assessed the level of self-efficacy and its corresponding prognostic factors among RA patients. Summarizing the results of the study, the authors state that healthcare providers need to assess the level of self-efficacy—a feature that seems to be related to a few medical characteristics—to facilitate the adaptation of healthcare regimens [5].

Anti-neutrophil cytoplasmic antibody (ANCA)-associated vasculitis (AAV) targets small and medium-sized blood vessels and is hallmarked by ANCA production. The term “ANCA-negative” is applied if a person fulfills the criteria for AAV but has negative serological ANCA test findings. Deresevičienė G. et al. conducted a cross-sectional study at a tertiary university hospital involving 73 patients diagnosed with anti-neutrophil cytoplasmic antibody (ANCA)-associated vasculitis (AAV) to evaluate clinical features of ANCA-positive and ANCA-negative vasculitis patients. The results of the study showed that the clinical condition of ANCA-positive patients is more severe in terms of organ damage and laboratory changes [6].

Children with juvenile idiopathic arthritis (JIA) suffer from a reduced quality of life due to their illness, and there is little research on child and parent satisfaction with disease management. Using symptom status acceptable to parents of children with juvenile arthritis (JA-PASS), demographic characteristics, disease course, and treatment, Romano F et al. conducted a cross-sectional study of 63 children diagnosed with JIA to investigate factors influencing parental satisfaction with the management of their children’s JIA. Summarizing the results of the study, the authors conclude that JA-PASS provides useful information on how parents see the disease progressing and how well treatments work, and they suggest adding it to patient management [7].

There is insufficient data on immune disorders in systemic lupus erythematosus (SLE), especially those associated with disease flares. Kosałka-Węgiel J et al. revealed significant links between different B cell subsets and SLE disease activity. The authors emphasize an individualized approach to SLE patients in order to identify those at higher risk of SLE flare-ups [8].

Viral infections, including coronavirus disease 2019 (COVID-19), tend to cause more severe disease in patients with autoimmune rheumatic diseases (AIRD). Rutskaya-Moroshan K et al. examined the clinical characteristics of rheumatology patients and risk factors for severe infection. AIRDs of the study included rheumatoid arthritis, ankylosing spondylitis, systemic lupus erythematosus, and systemic sclerosis. This study showed that patients with AIRD were more likely to experience joint pain, depression, and shortness of breath. Patients with AIRD tended to have more severe diseases. Patients with arterial hypertension, diabetes mellitus, chronic kidney and lung diseases, receiving corticosteroid treatment, had longer illnesses, and highly active autoimmune disease increased the chance of getting a more severe form of COVID-19 [9].

Review by Vaduva O-A et al. demonstrated that dietary choices can lead to significant modification Psoriasis Area and Severity Index (PASI) scores. Low-calorie diets rich in antioxidants are effective, especially when the focus is on vegetables and animal proteins are restricted. The Mediterranean diet and possibly the ketogenic diet are beneficial because they provide omega-3 fatty acids and may alter the gut microbiome. Topical vitamin D and its analogs, together with corticosteroids, can alleviate psoriasis symptoms at the skin level. Low-calorie diets containing antioxidants are very powerful in this regard, especially when the focus is on vegetables and the consumption of animal-derived proteins is limited. Oral vitamin D supplements have a positive effect on psoriatic arthritis and may reduce the risk of related complications [10].

In order to properly assess the systemic manifestations of Sjögren’s disease and to individualize the treatment of Sjögren’s disease, the article by Mieliauskaitė D and Kontenis V presented evidence-based links between Sjögren’s disease and gastroesophageal reflux disease (GERD). In general, there is a limited number of evidence-based studies evaluating the relationship between GERD and Sjögren’s disease and GERD-related alterations in the microbiota in a multidisciplinary context. Future studies are necessary to improve the timely diagnosis and individualized treatment of Sjögren’s disease [11].

Fibroblast-like synoviocytes (FLS) are one of the main factors in most cases of arthritis. Kirdaite G. et al. review the hallmarks and therapeutic challenges at the time of arthritis course, and review novel treatment targets, including surface biomarkers and intracellular proteins, non-coding ribonucleic acids (RNA) [12].

The review by Alexandru C et al. attracted considerable interest. This review examines the role of anti-receptor recognition particle (anti-SRP) antibodies in the diagnosis and treatment of collagen diseases with cardiac damage.

Rare reports of overlap syndrome between systemic sclerosis and immune-mediated necrotizing myositis have been reported. It is known that myositis can be a sign of scleroderma, and it is important to diagnose the overlap syndrome of these two diseases, as they can be interpreted as separate diseases, although specific treatment and monitoring should be applied. Anti-signal recognition particle (anti-SRP) antibodies act on skeletal muscle fibers and suppress myoblast regeneration, which leads to muscle fiber atrophy and necrosis. Anti-SRP antibodies are characteristic of immune-mediated necrotizing myopathies, which are characterized by muscle necrosis and minimum inflammatory response, with weakness of proximal muscles and representative external muscle symptoms. There is conflicting evidence regarding the link between cardiac symptoms and the presence of these antibodies, and the latest studies cannot prove a meaningful relationship between these two factors. Myocarditis is a condition with unpredictable and possibly serious consequences, ranging from heart failure and dilated cardiomyopathy to death. Diagnosing this disease is difficult because myocardial biopsy is an invasive procedure, so the results of cardiac magnetic resonance imaging are usually evaluated to make assumptions. This article addressed issues related to the management of collagen diseases by investigating the role of anti-SRP antibodies in the pathogenesis of cardiac damage [13].

Challenges remain in the field of rheumatology due to an aging population and the complexity of diseases, the need to manage comorbidity, the need to address health disparities between different population groups, and the challenges associated with the effective and appropriate integration of new technologies into the management of autoimmune rheumatic diseases.
